# Twist and snai1 expression in pharyngeal squamous cell carcinoma stroma is related to cancer progression

**DOI:** 10.1186/1471-2407-11-350

**Published:** 2011-08-11

**Authors:** Anna Jouppila-Mättö, Mervi Närkiö-Mäkelä, Ylermi Soini, Matti Pukkila, Reijo Sironen, Hanna Tuhkanen, Arto Mannermaa, Veli-Matti Kosma

**Affiliations:** 1 Department of Otorhinolaryngology - Head and Neck Surgery, Kuopio University Hospital, P.O.Box 1777, FI-70211 Kuopio, Finland; 2 Institute of Clinical Medicine, Otorhinolaryngology - Head and Neck Surgery, University of Eastern Finland, P.O.Box 1627, FI-70211 Kuopio, Finland; 3 Institute of Clinical Medicine, Pathology and Forensic Medicine, University of Eastern Finland, P.O.Box 1627, FI-70211 Kuopio, Finland; 4 Department of Clinical Pathology, Kuopio University Hospital, P.O.Box 1777, FI-70211 Kuopio, Finland; 5 Biocenter Kuopio and Cancer Center of Eastern Finland, University of Eastern Finland, P.O.Box 1627, FI-70211 Kuopio, Finland

**Keywords:** Pharyngeal squamous cell carcinoma, Stromal cells, TWIST, SNAI1, Prognosis, Epithelial-mesenchymal transition

## Abstract

**Background:**

Epithelial-mesenchymal transition (EMT) is a crucial process in tumorigenesis since tumor cells attain fibroblast-like features enabling them to invade to surrounding tissue. Two transcription factors, *TWIST *and *SNAI1*, are fundamental in regulating EMT.

**Methods:**

Immunohistochemistry was used to study the expression of TWIST and SNAI1 in 109 pharyngeal squamous cell carcinomas.

**Results:**

Tumors with intense stromal staining of TWIST relapsed more frequently (p = 0.04). Tumors with both positive TWIST and SNAI1 immunoreactivity in the stroma were at least Stage II (p = 0.05) and located more often in hypopharynx (p = 0.035). Tumors with negative immunostaining of TWIST and SNAI1 in the stromal compartment were smaller (T1-2) (p = 0.008), less advanced (SI-II) (p = 0.031) and located more often in the oropharynx (p = 0.007). Patients with negative SNAI1 and TWIST immunostaining in tumor stroma had a better 5-year disease-specific and overall survival (p = 0.037 and p = 0.014 respectively).

**Conclusion:**

TWIST and SNAI1 expression in stromal cells is associated with clinical and histopathological characteristics that indicate progressive disease. Negative expression of these EMT-promoting transcription factors predicts a better outcome.

## Background

Head and neck cancer, over 90% of which are squamous cell carcinomas (SCC) [1], is the sixth most common malignancy and eighth leading cause of cancer death worldwide [2]. The prognosis of pharyngeal squamous cell carcinoma (PSCC) is the poorest of all head and neck cancers and the survival rate has not improved significantly in the last two decades [3]. The delay in making a diagnosis, the appearance of second primaries, as well as regional and distant metastases all contributes to the poor survival [2].

Carcinogenesis is a multistep process characterized by the gradual accumulation of mutations in cancer cells. However, cancer cells also modify their stromal surroundings to create a supportive environment to permit tumor progression.

The tumor stroma is the compartment providing the connective-tissue framework of the tumor. This includes fibroblasts, immune and inflammatory cells, fat cells and blood-vessels. Normal fibroblasts are able to inhibit cancer progression. But during early tumor development, however, there are changes in the local tissue microenvironment which shifts to growth-promoting stage due to local conditions such as chronic inflammation [4]. Moreover, cancer associated fibroblasts have been shown to be important promoters of tumor growth and progression [5].

Epithelial-mesenchymal transition (EMT) is a complex process during which cellular phenotype and function become changed towards a migrating and invasive form. This process is crucial in embryogenesis. But, it is also frequently seen in tumor progression [6]. It is triggered by a diverse set of stimuli e.g. growth-factor signaling, hypoxia and also by tumor-stromal cell interactions with transcription factors such as *SNAI1, TWIST, SLUG *and *ZEB1 *[7]. One important property of *TWIST *and *SNAI1 *is the direct repression of E-cadherin, resulting in a loss of cell-cell adhesion [8,9]. *TWIST *and *SNAI1 *have also antiapoptotic properties [10,11] they influence cell polarity [12], and angiogenesis [13].

*TWIST *is a helix-loop-helix transcription factor, which induces motility and metastatic phenotype in cell lines [14,15]. High expression of TWIST is associated with aggressive tumor properties and poor survival in oesophageal and cervical SCC [16,17]. In our previous study, it was found that the endothelial and stromal staining of SNAI1 was associated with poor survival in PSCC [18]. In a recent study of oral SCC a small group of cases with abundant SNAI1 expression in tumor cell nuclei had significantly shorter DSS [19]. In head and neck squamous cell carcinoma (HNSCC), Yang et al have shown that co-expression of TWIST and SNAI1 was associated with a shorter metastasis-free period and reduced overall survival [20]. The prognosis of cases with TWIST and SNAI1 co-expression is also worse in hepatocellular carcinoma and non-small cell lung cancer compared with cases where only one of the transcriptional factors is expressed [15,21].

According to our hypothesis TWIST and SNAI1 could affect survival in PSCC. Previous studies have focused mainly on nuclear and cytoplasmic expression of TWIST and SNAI1 in epithelial cancer cells, not on the whole tumor microenvironment. Furthermore, there are only a few studies into TWIST expression in HNSCC. The aim of present study was to separately evaluate stromal and epithelial cancer cell expression of TWIST in PSCC cohort and its co-expression with SNAI1. We also wanted to investigate the association of these transcription factors with clinicopathological features and their impact on patient survival over the long-term.

## Methods

### Patients

The original clinical data from hospital records included all patients from eastern Finland diagnosed with oro- or hypopharyngeal squamous cell carcinoma between years 1971 and 1997 (n = 138). Sufficient material for immunohistochemical analyses was available from 109 original tumor samples and the representativeness of the groups was confirmed by χ^2^-test [22]. The histological differentiation was evaluated according to the World Health Organization (WHO) classification [23] and the tumor staging was based on the Classification of the International Association Against Cancer (UICC) [24]. The Karnofsky performance status at the time of diagnosis was scored [25]. The patients were monitored until death or April 2009 and none of the patients was lost to follow-up.

### Tissue microarray and immunohistochemistry

The most representative areas of each tumor were chosen by an experienced pathologist (YS) and marked for inclusion into tissue microarrays that were constructed using 1.0 mm core Manual tissue arrayer I (Beecher Instruments, Silver Spring, MD, USA). Four-μm-thick sections were first deparaffinized and rehydrated in the routine manner. Then the sections for TWIST analysis were heated in a microwave oven (800 W) for 3 × 5 min in citrate buffer (pH 6.0) and sections for SNAI1 analysis in Tris-EDTA buffer (pH 9.0), incubated in that buffer for 18 min and washed twice for 5 min in phosphate buffered saline (PBS). Endogenous peroxidase activity was blocked with hydrogen peroxide (5%, 5 min) followed by washing with water 2 × 5 min and with PBS for 2 × 5 min. Non-specific binding was blocked with 1.5% normal serum in PBS for 25 min at room temperature. The sections were incubated over night at 4°C with a mouse monoclonal antibody against TWIST (1:100 dilution) (Abcam, Cambridge, UK) and a mouse monoclonal antibody against SNAI1 (1:750 dilution) [26,27] respectively. In the negative controls, the primary antibody was omitted. The slides were then washed with PBS for 2 × 5 min and incubated with a biotinylated secondary antibody (ABC Vectastain Elite Kit, Vector Laboratories, Burlingame, CA, USA) for 35 min at room temperature. Subsequently, the slides were washed twice with PBS for 5 min, incubated for 45 min in preformed avidin-biotinylated peroxidase complex (ABC Vectastain Elite Kit, Vector Laboratories, Burlingame, CA, USA) and washed with PBS for 2 × 5 min. The color was developed with diaminobenzidine tetrahydrochloride (DAP) (Sigma, St. Louis, MO, USA). The samples were counterstained with Mayer's haematoxylin, washed, dehydrated, cleared and mounted with Depex (BDH, Poole, UK). Pharyngeal tumor tissue and ovarian tumor tissue with known positive TWIST and SNAI1 stainings were used as positive controls.

### Evaluation of the expression pattern

Both TWIST and SNAI1 array spots were initially evaluated separately by three observers (AJ-M, RS, YS and AJ-M, HT, YS respectively) without any knowledge of the clinical data. The results were compared and in case of a disagreement, the case was re-evaluated by all investigators in order to reach a consensus. In TWIST array spots the stromal and epithelial cancer cell immunoreactivity was classified into 5 categories according to the proportion of positive cell nuclei as follows; 1 = 0-4%, 2 = 5-25%, 3 = 26-50%, 4 = 51-75%, 5 = 76-100%. In SNAI1 array spots the immunoreactivity in stromal and epithelial cancer cells were counted on a continuous scale. The tumors were divided to TWIST and SNAI1 positive and negative cases according to the median of the evaluations (1 in epithelial and 2 in stromal cells for TWIST; 2 in epithelial and 3 in stromal cells for SNAI1).

### Statistical analyses

The chi-squared test was used in analyzing frequency tables of two variables. Frequency tables with three variables were calculated with one-way ANOVA -test. A Mann-Whitney test was used to examine the associations between continuous variables. The association between TWIST and SNAI1 staining was evaluated by Spearman test. Univariate survival analyses were evaluated using the Kaplan-Meier method. The statistical differences between the curves were analyzed using the log-rank test. Multivariate survival analysis was calculated using Cox's proportional hazards model. Disease-specific survival was defined as the time between the date of primary diagnostic biopsy and the date of death due to pharyngeal cancer. SPSS 14.0 software (SPSS Inc., Chicago, IL, USA) was used for statistical analyses.

### Ethics

The research plan was approved by the ethical committee of Kuopio University and Kuopio University Hospital and permission for accessing data from the Finnish Cancer Registry and from hospital records was obtained from by the Finnish Ministry of Social Affairs and Health.

## Results

### Cohort

The patients and treatment characteristics are summarized in Table 1. The mean age of the patients at the time of diagnosis was 65 years with a male predominance. Almost 70% of the carcinomas were advanced stages (III-IV) and 76% had a moderate to poor degree of differentiation. The most common primary treatment modality was radiotherapy either alone or postoperatively.

**Table 1:** Clinicopathological features of the patients with pharyngeal squamous cell carcinoma (*n *= 109)

*Variable*	*n (%)*
Mean age at the time of presentation, years	65 [40-89]*
Median duration of the symptoms, months	3 [0-76]*
Sex	
Male	82 (75)
Female	27 (25)
Site of primary tumor	
Oropharynx	69 (63)
Hypopharynx	40 (37)
T category	
T1	13 (12)
T2	40 (37)
T3	21 (19)
T4	35 (32)
N category	
N0	63 (58)
N1	16 (15)
N2	27 (25)
N3	3 (3)
M category	
M0	105 (96)
M1	4 (4)
Stage	
S I	9 (8)
S II	25 (23)
S III	21 (19)
S IV	54 (50)
Histologic differentiation	
Well	26 (24)
Moderate	48 (44)
Poor	35 (32)
Karnofsky performance status score	
≥ 70%	72 (66)
< 70%	37 (34)
Primary treatment	
Radiotherapy	69 (63)
Surgery and radiotherapy	31 (28)
Surgery	5 (5)
No cancer specific treatment	4 (4)
Recurrence	
No	37 (34)
Yes	41 (38)
No response	31 (28)
Second primary tumor	
No	99 (91)
Yes	10 (9)
Median OS, months	20.9 [1.1-401.3]*

*Values in square brackets indicate range.

### Expression of TWIST

**Figure 1:**
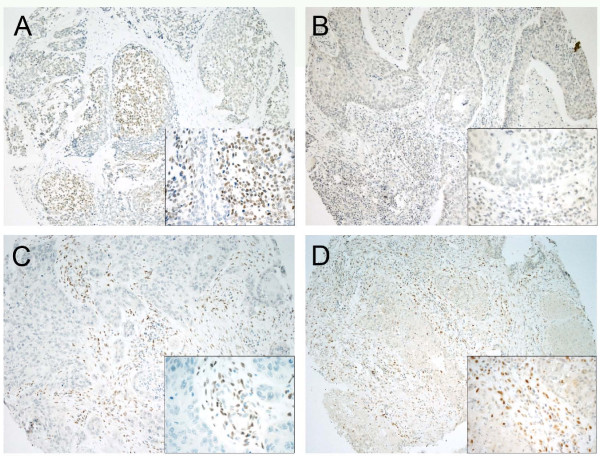
**Expression of TWIST and SNAI1 nuclear transcription factors in pharyngeal squamous cell carcinoma**. Positive (A) and negative (B) epithelial TWIST immunostaining in squamous epithelial cells (immunoscores 3/5 and 0/5, respectively). Positive stromal TWIST staining in stromal spindled cells (C; immunoscore 4/5). Positive SNAI1 immunostaining in the spindled stromal cells (D; immunoscore > 30 cells). Tumors A and D are moderately differentiated (grade 2/3) and tumours B and C poorly differentiated (grade 3/3). The stages are IV (A), II (B), IV (C) and IV (D). (Original magnifications of x100 in low power views and × 400 in the inserts).

TWIST staining was detected in the cell nuclei of stromal fibroblasts and epithelial cancer cells (Figure 1). The stromal expression of TWIST was clearly more common than its expression in the epithelial tumor cell compartment. According to the median (> = 2), 44 of 109 tumors (40%) were classified as being stroma positive. According to median (> = 1), 38 epithelial compartments of the tumors out of 109 (35%) were classified as TWIST positive. The percentage of positive immunostaining of TWIST in the cell nuclei of epithelial and stromal cells is detailed in Table 2.

**Table 2:** Proportion of TWIST expression in cell nuclei of epithelial cancer cells and stromal fibroblasts

Category	TWIST expression %	Epithelial cancer cells n (%)	Stromal cells n (%)
1	0-4	78 (71)	28 (26)
2	5-25	19 (18)	41 (37)
3	26-50	4 (4)	24 (22)
4	51-75	7 (6)	14 (13)
5	≥ 76	0 (0)	1 (1)
nd*	nd	1 (1)	1 (1)

*nd = not defined

### Clinicopathological variables and TWIST expression

Tumors with abundant TWIST expression in stromal fibroblast cell nuclei (category 3 or more in both samples) relapsed more frequently (p = 0.04). Stromal staining was more common in hypopharyngeal than oropharyngeal tumors (p = 0.015). Stromal nuclear expression of TWIST did not associate with the patient's age, gender, tumor size, stage, histological grade or smoking habits. However, there was a tendency for an association between stromal TWIST immunoreactivity and larger tumor size (T3-4) and advanced stage (SIII-IV) (p = 0.064 and p = 0.086 respectively). In our cohort, epithelial cancer cell staining of TWIST did not associate significantly with any of the clinicopathological variables. However, patients with epithelial cancer cell immunoreactivity tended to have a worse Karnofsky performance status (score < 70; p = 0.077) and also to display neck lymph node metastases (p = 0.099).

### TWIST and SNAI1 co-expression

SNAI1 expression has been described in detail in our previous study with the same patient material [18]. Epithelial cancer cell immunoreactivity of the nuclei was present in 75 (68%) with stromal immunoreactivity in 49 (46%) of the array spots (Figure 1). A strong association between TWIST and SNAI1 expression in stromal cell nuclei was detected (p = 0.001, Spearman's correlation coefficient 0.33). But no association was detected in the epithelial cancer cell nuclei (p = 0.206, Spearman's correlation coefficient 0.10).

To evaluate tumors undergoing TWIST and SNAI1 related EMT we selected a group, where both TWIST and SNAI1 were positive and renamed it EMT+ (n = 29, 27%). All these EMT+ tumors were at least Stage II (p = 0.05) and were located more often in hypopharynx (p = 0.035). TWIST and SNAI1 co-expression did not correlate with histological grade, metastases or smoking habits. Correspondingly, tumors lacking both TWIST and SNAI1 stromal immunoreactivity (n = 42, 38.5%) were renamed EMT-. These tumors were smaller (T1-2) (p = 0.008), less advanced (SI-II) (p = 0.031) and located more often in oropharynx (p = 0.007). Furthermore, non-smokers had more EMT- tumors (p = 0.034). It was possible to detect either SNAI1 or TWIST stromal immunoreactivity in thirty eight (35%) patients. Tumors were larger according to the number of positive transcription factors (p = 0.034) (Table 3). It was estimated that tumors were also more often located in hypopharynx when both transcription factors were positive (p = 0.023) (Table 4).

**Table 3:** Expression of TWIST and/or SNAI1 in tumor stroma in different T categories

	Stromal expression of TWIST and/or SNAI1			
T classification	EMT-n (%)	TWISTor SNAI1 + n (%)	EMT+n (%)	*p*
T1 and T2	27 (25)	15 (14)	11 (10)	0.034
T3 and T4	15 (14)	23 (21)	18 (16)	

**Table 4:** Expression of TWIST and/or SNAI1 in tumor stroma in different sites

	Stromal expression of TWIST and/or SNAI1			
Site	EMT-n (%)	TWIST or SNAI1 + n (%)	EMT+n (%)	*p*
Oropharynx	33 (30)	22 (20)	14 (13)	0.023
Hypopharynx	9 (8)	16 (15)	15 (14)	

### Survival analyses

**Figure 2:**
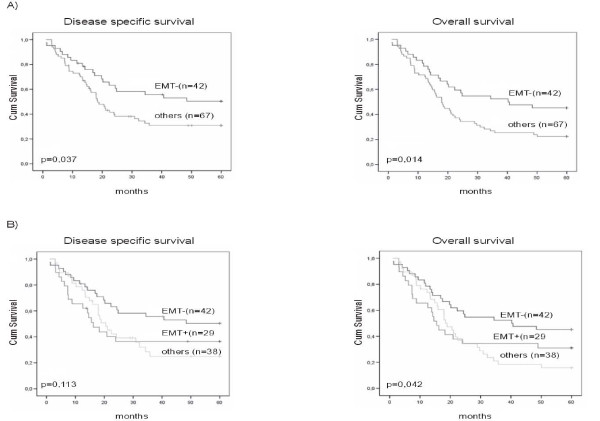
**Kaplan-Meier univariate 5-year survival analysis in pharyngeal squamous cell carcinoma**. In the EMT- group both TWIST and SNAI1 were negative and in EMT+ group both transcription factors were positive. In group others, both or either TWIST or SNAI1 were expressed in tumor stroma. Absence of TWIST and SNAI1 expression in tumor stroma predicted better DSS and OS A). Survival was poorer when one or both transcription factors were positive in tumor stroma B).

In Kaplan-Meier univariate analysis neither stromal nor epithelial cancer cell expression of TWIST alone associated with DSS or OS. Tumors with both TWIST and SNAI1 negativity had a statistically significantly better 5-year survival compared with patients who displayed positive TWIST and/or SNAI1 stromal cell nuclei staining (DSS p = 0.037; OS p = 0.014) (Figure 2a). The 5-year prognosis tended to worsen depending on the number of positive transcription factors detected (DSS p = 0.113; OS p = 0.043) (Figure 2b). In the Cox proportional hazards model, tumor stage and Karnofsky performance status score were the only independent prognostic factors for survival (p < 0.001 in both).

## Discussion

During EMT, epithelial cells attain mesenchymal and promigratory features and are able to become detached from the epithelial layer. EMT is also a crucial property during morphogenesis. But, it has also a central role in tumor progression. Different transcription factors such as *SNAI1 *and *TWIST *regulate gene expression patterns that underlie EMT [6]. In the present study, tumors that did not express TWIST or SNAI1 in stroma were smaller, of lower stage and were more often situated in the oropharynx. Tumors with negative stromal immunostaining for TWIST and SNAI1 had a significantly better 5-year DSS. Interestingly, the patients with stromal TWIST and SNAI1 negative tumors were more often non-smokers. In bladder cancer TWIST expression has also been shown to be influenced by smoking [28]. This agrees with a previous hypothesis that smoking can modulate the expression of EMT markers. Aromatic hydrocarbon B[a]P in tobacco smoke has shown to cause EMT-like, partly irreversible dynamic changes in gene expression such as TWIST up-regulation [29].

In the present study, nuclear staining intensity of TWIST was detected in the stromal component more often than in epithelial cancer cells. The same phenomenon was recently observed with SNAI1 in oral SCC [19]. There was also a trend to more pronounced TWIST staining intensity in larger and advanced stage tumors. Stromal expression of TWIST was more frequent in hypopharyngeal than in oropharyngeal tumors. It remains unclear whether this is due to more advanced disease stage at the time of diagnosis or it represents a true feature of the hypopharyngeal tumors with very poor overall prognosis.

TWIST has several properties that facilitate tumor progression including the triggering of EMT, inhibition of apoptosis and the enhancement of angiogenesis [9,13]. TWIST has shown to induce EMT through chromatin remodeling [30]. TWIST overexpression correlates with E-cadherin downregulation in HNSCC cell line [30]. However, in a recent study of nasopharyngeal carcinoma SNAI1 expression associated with repressed E-cadherin expression while TWIST expression had no affect on expression of E-cadherin [31]. Thus, EMT regulators may show varied expression and roles in different kinds of carcinomas.

To date, there is only one other study which has examined the association between Twist expression and clinicopathological features in HNSCC. In a rather small cohort of various head and neck squamous cell carcinomas (n = 50), nuclear and cytoplasmic staining of TWIST correlated with differentiation grade, advanced stage and large neck lymph node metastases [32]. The comparison of these data with this present study has to be conducted with care. Since transcription factors are considered to be active only in the cell nuclei [33], cytoplasmic staining was not included into our evaluation. In the present study, TWIST expression alone didn't associate with survival. There is some evidence of positive staining of TWIST and poor survival in HNSCC and for example in oesophageal SCC [20,16]. In addition, TWIST expression in the epithelial compartment of breast carcinoma was associated with poor survival [34]. Similarly to the situation in breast carcinoma, nuclear staining of TWIST in epithelial cancer cells was uncommon with two thirds of tumors lacking immunoreactivity [34]. A similar observation was seen also in the recent study of HNSCC [20]. In oesophageal SCC and cervical SCC, TWIST immunoreactivity was more abundant [16,17]. Evidently, tumors of different sites and histology vary in their expression of TWIST. There is also variation between classifying strategies in different publications such as diverge cut points between positive and negative values and in some studies also staining intensity has been evaluated.

In the present study, patients with epithelial cell nuclei expression of TWIST tended to have more neck lymph node metastases. In breast tumor cell lines, TWIST has been shown to promote early steps of tumor metastasis, where tumor cells gain access to circulation [14]. TWIST expression also correlates with distant metastases for example in esophageal SCC [35]. However, the metastatic event is a complex process with many phases and there are several other factors which regulate it.

There are no published data about the half-life of TWIST or on its stability in cell nucleus but SNAI1 has shown to be highly unstable [33]. As is typical for transcription factors it is probable that TWIST is expressed in tumor cells for only a very short time which might explain why only one third of the epithelial cancer cells expressed TWIST in their cell nucleus. Some of the stromal cells may represent transformed epithelial cancer cells undergoing EMT [7]. Tumor cells undergoing EMT lose their epithelial characteristics, and a proportion of stromal cells might represent transformed tumor cells. Furthermore, as a response to growth factors, transcription factors might stimulate the conversion of stromal fibroblasts into more motile myofibroblasts [36]. There are also non-neoplastic activated fibroblastic cells in tumor stroma which may express transcription factors via the interaction with epithelial cancer cells. These stromal cells might also express TWIST or SNAI1 and cannot be distinguished by immunohistochemistry alone from EMT transformed stromal cells.

Elevated nuclear transcription factor expression of TWIST and SNAI1 in tumor stroma may be evidence of ongoing EMT in pharyngeal squamous cell carcinoma. In line with this, tumors with negative transcription factor expression were smaller and had a better prognosis than tumors with positive expression. Ongoing EMT can thus be related to tumor progression in pharyngeal squamous cell carcinoma.

The prognosis of PSCC tended to worsen in conjunction with the increased stromal expression of these transcription factors. Other studies with TWIST and SNAI1 also support the hypothesis that these two transcription factors act in collaboration to promote EMT [15,19]. Even though they are regulated independently [15]. In the present study only advanced stage and a high Karnofsky performance status score were independent prognostic factors in the multivariate analysis. However, co-expression of the transcription factors, a sign of undergoing EMT, tended to associate with larger tumor size and more advanced stage. There is evidence of complex communication taking place between tumor cells and their microenvironment [4]. The transcription factors involved in EMT promotion are induced in cancer cells in response to signal released by activated stroma [9].

## Conclusion

In summary, we showed that the TWIST and SNAI1 expression in stromal cells is associated with histopathological and clinical characteristics that indicate progressive disease. Negative expression of these EMT-promoting transcription factors predicts a better outcome. These findings emphasize the importance of the tumor microenvironment in enhancing cancer growth and facilitating metastasis.

## List of Abbreviations

EMT: epithelial-mesenchymal transition; HNSCC: head and neck squamous cell carcinoma; DSS: disease specific survival; OS: overall survival; PBS: phosphate buffered saline; PSCC: pharyngeal squamous cell carcinoma; SCC: squamous cell carcinoma

## Competing interests

The authors declare that they have no competing interests.

## Authors' contributions

AJ-M, HT, YS and RS carried out the IHC analysis. AJ-M, MN-M, MPU, V-MK and AM participated in study design and statistical analysis. AJ-M and MN-M drafted the manuscript. All authors read and approved the final manuscript.

## Pre-publication history

The pre-publication history for this paper can be accessed here:

http://www.biomedcentral.com/1471-2407/11/350/prepub
